# The chromatin modification by SUMO-2/3 but not SUMO-1 prevents the epigenetic activation of key immune-related genes during Kaposi’s sarcoma associated herpesvirus reactivation

**DOI:** 10.1186/1471-2164-14-824

**Published:** 2013-11-23

**Authors:** Pei-Ching Chang, Chia-Yang Cheng, Mel Campbell, Yi-Cheng Yang, Hung-Wei Hsu, Ting-Yu Chang, Chia-Han Chu, Yi-Wei Lee, Chiu-Lien Hung, Shi-Mei Lai, Clifford G Tepper, Wen-Ping Hsieh, Hsei-Wei Wang, Chuan-Yi Tang, Wen-Ching Wang, Hsing-Jien Kung

**Affiliations:** Institute of Microbiology and Immunology, National Yang-Ming University, Taipei, 11221 Taiwan; Institute of Molecular and Cellular Biology and Department of Life Science, National Tsing Hua University, Hsinchu, 300 Taiwan; Department of Computer Science, National Tsing Hua University, Hsinchu, 300 Taiwan; UC Davis Cancer Center, University of California, Davis, CA 95616 USA; Division of Molecular and Genomic Medicine, National Health Research Institutes, 35 Keyan Road, Zhunan, Miaoli County, 35053 Taiwan; Department of Biochemistry and Molecular Medicine, University of California, Davis, CA 95616 USA; Institute of Statistics, National Tsing Hua University, Hsinchu, 300 Taiwan; Institute for Translational Medicine, College of Medical Science and Technology, Taipei Medical University, 250 Wu-Xin Street, Taipei City, Taiwan

## Abstract

**Background:**

SUMOylation, as part of the epigenetic regulation of transcription, has been intensively studied in lower eukaryotes that contain only a single SUMO protein; however, the functions of SUMOylation during mammalian epigenetic transcriptional regulation are largely uncharacterized. Mammals express three major SUMO paralogues: SUMO-1, SUMO-2, and SUMO-3 (normally referred to as SUMO-1 and SUMO-2/3). Herpesviruses, including Kaposi’s sarcoma associated herpesvirus (KSHV), seem to have evolved mechanisms that directly or indirectly modulate the SUMO machinery in order to evade host immune surveillance, thus advancing their survival. Interestingly, KSHV encodes a SUMO E3 ligase, K-bZIP, with specificity toward SUMO-2/3 and is an excellent model for investigating the global functional differences between SUMO paralogues.

**Results:**

We investigated the effect of experimental herpesvirus reactivation in a KSHV infected B lymphoma cell line on genomic SUMO-1 and SUMO-2/3 binding profiles together with the potential role of chromatin SUMOylation in transcription regulation. This was carried out via high-throughput sequencing analysis. Interestingly, chromatin immunoprecipitation sequencing (ChIP-seq) experiments showed that KSHV reactivation is accompanied by a significant increase in SUMO-2/3 modification around promoter regions, but SUMO-1 enrichment was absent. Expression profiling revealed that the SUMO-2/3 targeted genes are primarily highly transcribed genes that show no expression changes during viral reactivation. Gene ontology analysis further showed that these genes are involved in cellular immune responses and cytokine signaling. High-throughput annotation of SUMO occupancy of transcription factor binding sites (TFBS) pinpointed the presence of three master regulators of immune responses, IRF-1, IRF-2, and IRF-7, as potential SUMO-2/3 targeted transcriptional factors after KSHV reactivation.

**Conclusion:**

Our study is the first to identify differential genome-wide SUMO modifications between SUMO paralogues during herpesvirus reactivation. Our findings indicate that SUMO-2/3 modification near protein-coding gene promoters occurs in order to maintain host immune-related gene unaltered during viral reactivation.

## Background

SUMOylation was initially identified as a reversible post-translational modification that controls a variety of cellular processes, including cellular signal transduction, replication, chromosome segregation, and DNA repair [1–3]. The growing list of Small Ubiquitin-like MOdifier (SUMO) substrates includes transcription factors and epigenetic regulators, which implies the involvement of the SUMO modification system in the epigenetic regulation of gene expression [4] and in the initiation and maintaining of heterochromatin silencing [5, 6]. SUMO has been found in all eukaryotes but is not present in prokaryotes. The global regulatory role of SUMOylation in gene expression and protein interaction has been richly explored in lower eukaryotes such as yeast [7, 8]. However, there is only a single SUMO protein in yeast, whereas there are three major protein conjugating isoforms present in mammals; these are SUMO-1, and the highly similar SUMO-2 and SUMO-3, which are often refer to as SUMO-2/3. Recent reports have pinpointed some important differences between SUMO-1 and SUMO-2/3. These are, firstly, that SUMO-1 is conjugated to its substrates as a mono-SUMOylation, whereas SUMO-2/3 are able to form poly-SUMOylation chains [9]. Moreover, SUMO-1 acts like a chain terminator to the SUMO-2/3 polymers [10]. Secondly, inside cells, SUMO-1 appears mostly conjugated to proteins, whereas SUMO-2/3 are primarily found in the free form and are increased in conjugation to substrates when there are cellular stresses [11, 12]. Thirdly, the kinetics of SUMO-1 de-conjugation is slower than that of SUMO-2/3 [13]. Fourthly, a preferential association of SUMO-1 with the nuclear envelope and nucleolus, whereas SUMO-2/3 are distributed throughout the nucleoplasm [12]. Fifthly, although many substrates can be modified by both SUMO-1 and SUMO-2/3, some substrates are preferentially modified by one SUMO isoform or the other. The underlying complexity of SUMOylation has been extended by the identification of non-covalent interaction with effectors via SUMO interaction motifs (SIMs) [14]. SIMs are critical to both SUMO conjugation and SUMO-mediated effects. Structure analysis shows the potential differential specificity of SIMs toward SUMO paralogues [15]. The specificity of the SIM in relation to the SUMO E3 ligase [16–18] and substrate [19] has been found to control SUMO paralogue-specific modification. Consequentially, this provides an additional interaction platform for the selective recruitment of SUMO-1 or SUMO-2/3 specific SIM-containing effector proteins. While numerous studies have provided considerable insight into the differences in specificity between SUMO paralogues, their scope has been usually limited to a single host factor in each case. Discerning the genome-wide chromatin modification by SUMO paralogue during herpesvirus reactivation will greatly advance our knowledge of their differential role in epigenetic regulation and pathogenesis.

Due to the functional flexibility and far-reaching downstream consequences of SUMO, viruses have evolved different strategies that are able to manipulate the SUMO pathway and improve their survival [20–25]. This makes SUMO a potential target for antiviral therapy. Most current knowledge related to SUMO modification and viruses has been obtained from studying DNA tumor viruses, especially members of the *herpesviridae* and have been inevitably linked to counteracting the host’s antiviral properties. SUMOylation has been found to affect most of the immediate-early and early proteins of herpesviruses, which are usually transcriptional factors. BZLF1 and Rta of Epstein-Barr virus (EBV) [26–29], and the K-bZIP of KSHV are three such examples [25]. Viruses are also able to directly target the key enzymes of the SUMOylation pathway, namely the SUMO E1 activating enzyme, Aos1/Uba2, the SUMO E2 enzyme, Ubc9, the SUMO E3 ligases, and the SUMO protease SENP/Ulp; this allows the virus to take charge of the SUMOylation modulating factors in the cell [30]. Recently, we identified the first viral SUMO E3 ligase, KSHV K-bZIP; this enzyme has specificity toward SUMO-2/3 [16]. The encoding of a SUMO-2/3 specific viral SUMO E3 ligase by KSHV suggests that, potentially, KSHV is able to exploit the SUMO pathway to globally regulate viral and host transcriptional programs. This, in turn, implies that SUMO-2/3 may function in a manner that is distinct from SUMO-1 during viral reactivation.

KSHV, also known as human herpesvirus type 8, is a γ-herpesvirus associated with Kaposi’s sarcoma (KS), primary effusion lymphomas (PEL) and multicentric Castlemen’s disease [31]. It is one of the seven recognized human cancer viruses [32]. Like all herpesvirus, KSHV has distinct latent and lytic phases. Establishment of latency is a common property of herpesvirus in infected cells and is able to prevent their elimination by the host immune response, to maintain life-long infection, and to induce tumorigenesis [33, 34]. In order to establish infection and maintain latency, KSHV has acquired a series of different strategies that are able to limit innate antiviral responses and evade host immune surveillance, thus allowing the persistence of infection. For example, KSHV dedicates a large portion of its genome to encoding cellular homologues of host immune modulators and is able to express unique viral proteins that have immunomodulatory roles [35, 36]. For instance, KSHV-replication and transcriptional activator (K-Rta), an immediate early (IE) protein of KSHV, which is able to activate a wide spectrum of KSHV lytic genes and thereby alone can induce viral reactivation, has been found to block the interferon (IFN) pathway by targeting interferon regulatory factor (IRF) for degradation. The KSHV-encoded basic leucine zipper protein (K-bZIP), one of the earliest viral protein expressed right after K-Rta during acute infection and viral reactivation, has also been found to inhibit the IFN pathway by direct impeding IRF binding to the IFN promoter [37, 38]. The IFN pathway has also been found to be repressed by K-bZIP in a SUMOylation-dependent manner [39]. Moreover, recent studies have shown that SUMOylation of the IRFs occurs during viral infection and these changes are essential to allowing the virus to negatively regulate the IFN pathway [24, 40, 41]. Another strategy employed by herpesviruses such as HSV-1, the prototypical member of the *Herpesviridae*, is the complete suppression of cellular gene expression, a process termed host shutoff. This phenotype is found during lytic herpesviral infection and is believed to play an important role in establish herpesviral latency [42]. In HSV-1, the global shutoff of host gene expression occurs via two major and distinct inhibitory pathways. One is a global increase in the rate of mRNA degradation and the other is a virus-induced suppression of host mRNA synthesis [43]. For KSHV, mRNA degradation is performed by the host shutoff factor SOX [44]. The linking of SUMOylation to transcription repression and the finding that K-bZIP is a SUMO-2/3 specific E3 ligase led us to examine the possibility that there may be global silencing of host genes by K-bZIP. We have reported previously that K-bZIP, when overexpressed, was indeed a general gene-silencer [45].

To gain a better understanding of the differential functionality of SUMO-1 and SUMO-2/3 conjugation on chromatin in transcriptional regulation of host genes during KSHV reactivation, we performed a genome-wide mapping of chromatin modification by SUMO paralogues using ChIP-seq, a technology that allows the direct identification of all SUMO binding sites on the genome. Here, we demonstrate that the chromatin-binding patterns for SUMO-1 and SUMO-2/3 are very similar in the non-reactivated control cells. Interestingly, during viral reactivation, distinct dynamic chromatin-binding of SUMO paralogues was observed. We have demonstrated that the chromatin occupancy of SUMO-2/3 but not of SUMO-1 is significantly increased during viral reactivation and this enrichment is not randomly distributed. Enrichment occurs in promoter regions where transcription factors binds. Potential SUMO-2/3 target TFs on the chromatin were identified by annotating SUMO peaks in relation to putative transcription factor binding sites (TFBS) using the Transfac Matrix Database. Here, we provide the first comprehensive profile that compares the SUMO-1 and SUMO-2/3 landscapes in the human genome and predicts the relevant potential modifying TFs that bind to the chromatin. Previous findings from yeast study have shown that SUMO is globally associated with transcriptionally active genes [7, 46] and facilitates the shutting off of induced gene transcription [7]. This suggests that SUMO modification may also play a global role in transcription regulation in mammals. Large scale comparative analysis of ChIP-seq and transcriptome studies using RNA-seq in this study indicates that both SUMO-1 and SUMO-2/3 label the promoters of highly active genes in the non-reactivated control cells. However, during KSHV reactivation, the SUMO-2/3 modifications are greatly enriched in the promoters of highly active genes that show little change in gene expression.Together with previous findings from other studies, our results indicate that SUMO-1 and SUMO-2/3 may play similar roles in maintaining the expression of highly transcriptional active genes in non-reactivated cells. However, the enrichment of SUMO-2/3 at transcriptionally active genes that show no change in expressional level during viral reactivation suggests that SUMO-2/3, but not SUMO-1, ensures the steady-state expression of host genes without overt activation during viral reactivation. Consistent with studies exploring the functional analysis of SUMO paralogues in specific protein molecules such as Daxx [47], in the present study we demonstrate that there are distinct differences in the global roles of SUMO-1 and SUMO-2/3 in cells that are under stress, such as when there is herpesvirus reactivation.

## Methods

### Cell culture

The TREx-Flag(x3)-His(x3)-K-Rta BCBL-1 cell line (TREx-F3H3-K-Rta BCBL-1) and TREx-Flag(x3)-His(x3)-K-bZIP BCBL-1 cell line (TREx-F3H3-K-bZIP BCBL-1) were grown in RPMI 1640 containing 15% FBS, 50 μg/ml blasticidin and 100 μg/ml hygromycin (Invitrogen, Carlsbad, CA). To induce KSHV reactivation, 0.2 μg/ml doxycycline (dox) was added to the TREx-F3H3-K-Rta BCBL-1 cells. K-Rta expression was confirmed by Western blot analysis.

The shRNA cassette of SUMO-2 (5′-CACCGAGGCAGATCAGATTCCGAT TCGAAAATCGGAATCTGATCTGCCTC-3′) and SUMO-3 (5′-CACCGGATGAA TCTGTAACTTAACGAATTAAGTTACAGATTCATCC-3′) was inserted into pLenti4-H1/TO shRNA plasmid and introduced into TREx-F3H3-K-Rta BCBL-1 cells by lentiviral transduction. Cells were selected for 14 days by 300 μg/ml zeocine (InvivoGen, ant-zn-1) and purified by Ficoll. Knockdown efficiency of SUMO-2/3 by shRNA were tested by treated the cells with doxycycline (Dox) for 24 and 48 hours. TREx-F3H3-K-Rta-shSUMO-2/3 BCBL-1 cells were maintained as described for TREx-F3H3-K-Rta BCBL-1 and supplemented with 300 μg/ml zeocine.

### Plasmid construction

The full-length human IRF-1, IRF-2 and IRF-7 cDNAs were obtained by reverse-transcription polymerase chain reaction (RT-PCR) using an RNA sample isolated from BCBL-1 and using an Oligo(dT) primer. The primers 5′-AAACGGTCCGATGCCCATCACTCGGATG-3′ and 5′-AAACGGACCGCTACG GTGCACAGGGAAT-3′ were used for RT-PCR and cloning of IRF-1, the primers 5′-AAACGGTCCGATGCCGGTGGAAAGGATG-3′ and 5′-AAACGGACCGTTAA CAGCTCTTGACGCG-3′ were used for RT-PCR and cloning IRF-2, and the primers 5′-AAACGGTCCGATGGCCTTGGCTCCTGAG-3′ and 5′-AAACGGACCGCTAG GCGGGCTGCTCCAG-3′ were used for RT-PCR and cloning IRF-7. The IRF DNA fragment were then cloned into pcDNA3 and confirmed by DNA sequencing.

### Chromatin immunoprecipitation-sequencing (ChIP-Seq), ChIP-reChIP assay and real-time quantitative PCR (qPCR)

After 12 hours of K-Rta induction to allow KSHV reactivation in the TREx-F3H3-K-Rta BCBL-1 cell line, 1 × 10^7^ cells were harvested. ChIP assays were performed following the protocol described by the Farnham laboratory (provided at http://genomics.ucdavis.edu/farnham). ChIP-reChIP assays were performed by Re-ChIP-IT kit (Active Motif, Carlsbad, CA) following the manufacturer’s instruction. ChIP grade rabbit polyclone antibodies specific against SUMO-1 [Y299] (Abcam, ab32058) and against SUMO-2/3 (Abcam, ab3742), as well as rabbit non-immune serum IgG (Alpha Diagnostic International, San Antonio, TX), were used for the ChIP and ChIP-reChIP assays. Rabbit monoclone antibodies specific against IRF-7 (GeneTex, Irvine, CA) was used for the ChIP-reChIP assays.

For ChIP-Seq assay, ChIPed DNA was prepared from 5 × 10^7^ cells that had been resuspended in 30 ul of ddH_2_O and ChIP-seq library construction was then carried by following the sample preparation protocol from Illumina. Short reads (100 bp) from both ends (paired-end sequencing) of size-selected (400 bp) DNA fragments were selected and subjected to high throughput sequencing on an Illumina® Genome Analyzer_*II*_ System. The ChIP-Seq data was aligned onto the human genome hg19 build using UCSC. Around 6 × 10^7^ reads were mapped for each sample after filtering and quality control (QC) were carried out. In this study we used the enriched region detection method of Avadis NGS (Strand Scientific Intelligence, San Francisco, CA) to localize potential protein binding sites in order to delineated the SUMO-1 and SUMO-2/3 binding patterns.

The binding sites were verified by SYBR® Green Based qPCR using a CFX connect™ real-time PCR detection system (Bio-Rad, Richmond, CA). Specific primer sets were designed around the identified binding sites for this purpose.

### RNA-seq and RT-qPCR analysis

Total RNA was harvested using TRIzol reagent (Invitrogen, Carlsbad, CA) from TREx-F3H3-K-Rta BCBL-1 at 12 and 24 hours after K-Rta induced viral reactivation according to the manufacturer’s instructions. RNA-seq was conducted at the Sequencing Core of National Research Program for Genomic Medicine at National Yang-Ming University VYM Genome Research Center using an Illumina Genome Analyzer_*II*_. Sequencing reads were first trimmed with human ribosomal RNA sequences (28S, 18S, 5S, human ribosomal DNA complete repeating unit and mitochondrial ribosomal RNA) by Bowtie (version 1.0.0) with default parameters and then aligned the high quality reads to human reference genome hg19 using TopHat (version 2.0.8b) with Bowtie version 2.1.0 and samtools (version 0.1.9) with transcriptome information obtained from Ensembl Release 70 and NonCode v3.0. The transcript abundances were estimated in fragments per kilobase of transcript per million mapped reads (FPKM) by Cufflinks version 2.1.1. Genes from all three samples with FPKM > 0.05 were considered to be expressed and were used for the remaining analysis. Differential gene expression of the samples (K-Rta induction for 12 and 24 hours *vs*. control) was analyzed by comparing FPKM. For RT-PCR, 2 μg of total RNA was reverse-transcribed using SuperScript™ III First-strand synthesis system (Invitrogen) and Oligo-dT. qPCR was carried out based on the manufacturer’s protocol (iQ SYBR Green Supermix, Bio-Rad).

### Immunoprecipitation and western blot analysis

Transfected 293 T cells were collected in modified radioimmune precipitation assay (RIPA) buffer (50 mM Tris–HCl [pH 6.7], 1% NP-40, 0.25% sodium deoxycholate, 150 mM NaCl, 1 mM EDTA) supplemented with 1X protease inhibitor cocktail (Roche, Penzberg, Germany). TCLs were incubated with anti-FLAG M2-agarose (Sigma-Aldrich, St. Louis, MO) overnight at 4°C. Immune complexes were captured by protein A and protein G Sepharose beads. Beads were washed, and the bound proteins were analyzed by immunoblotting. Antibodies used for immunoblotting were anti-IRF-1 (Cell Signaling Technologies, Beverly, MA), anti-IRF-2 (Cell Signaling Technologies), anti-IRF-7 (GeneTex), anti-SUMO-2/3 antibodies.

## Results

### Global identification of the chromatin binding patterns of SUMO paralogues reveals that KSHV reactivation is associated with specific enrichment of SUMO-2/3

SUMO modifications of transcription regulatory proteins and chromatin modifying enzymes are linked to the epigenetic regulation of gene transcription. SUMO-1 and SUMO-2/3 have both common and distinct substrates, but their global functional roles in epigenetic regulation have not as yet been fully investigated. As mentioned earlier, DNA viruses have evolved different strategies that allow them to manipulate the SUMO pathway in a manner that helps their survival. We previously identified a KSHV lytic protein, K-bZIP, as a viral SUMO E3 ligase with specificity toward SUMO-2/3 [16]. Using KSHV as a model in conjunction with ChIP-seq to interrogate the binding sites of the various SUMO paralogues during viral reactivation, we hoped to distinguish the epigenetic regulatory role of the SUMO isoforms during viral replication. KSHV is a particularly attractive model as its reactivation can be switched on by the expression of a single K-Rta gene, and a well characterized Dox-inducible TREx-F3H3-K-Rta BCBL-1 cell line is available for this purpose. This study has the ability to pinpoint the global functional differences in terms of epigenetic regulation of the SUMO isoforms during viral pathogenesis.

**Figure 1:**
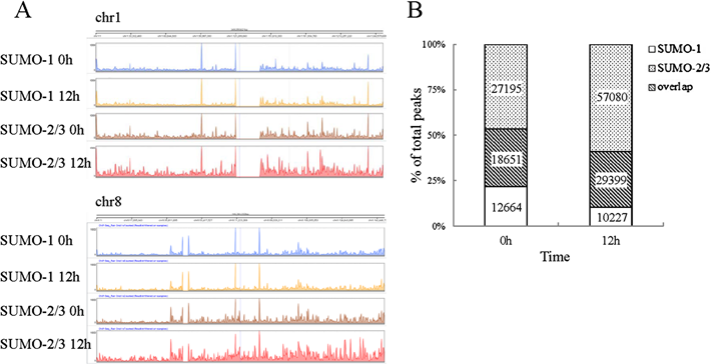
**Overview of ChIP-seq data showing chromatin occupancy of SUMO paralogues during KSHV reactivation. (A)** Histograms of ChIP-seq profiles across chromosome 1 and 8 for SUMO-1 and SUMO-2/3 binding sites before and after KSHV reactivation. **(B)** Overlap of SUMO-1 and SUMO-2/3 binding sites before and after KSHV reactivation in BCBL-1 cells; numbers indicate counts for overlapping and non-overlapping peaks.

To study the epigenetic regulation of SUMO paralogues in association with KSHV reactivation, the genome-wide *in vivo* binding sites of SUMO-1 and SUMO-2/3 were analyzed using massively parallel chromatin immunoprecipitation in combination with high throughput sequencing (ChIP-Seq); these processes were carried out on a K-Rta-inducible KSHV infected primary effusion lymphoma (PEL) cell line, TREx-F3H3-K-Rta BCBL-1. Chromatin samples from TREx-F3H3-K-Rta BCBL-1 cells before and after K-Rta induction to allow KSHV reactivation were isolated and subjected to the ChIP assay using ChIP grade SUMO-1 and SUMO-2/3 antibodies. High-throughput sequencing was then performed to measure the binding of SUMO-1 and SUMO-2/3 from a single run of ChIP assay. Approximately the same number (6 × 10^7^) of reads from the KSHV un-induced and induced samples were mapped to the human reference genome, hg19. Using an enrichment peak calling algorithm, we found a total of 31315 and 45846 high confidence SUMO-1 and SUMO-2/3 enrichment regions, respectively, in the non-reactivated control cells. After K-Rta induction for 12 hours, a total of 39626 SUMO-1 enrichment regions (an increase in 8 K peaks of ~1.3-fold compared to the control cells) and 86479 high confidence SUMO-2/3 enrichment regions (an increase in 40 k peaks of ~1.9-fold compared to the control cells) were identified (Figure 1A). Consistent with our previous findings showing that KSHV encodes a SUMO-2/3 specific E3 ligase in its lytic phase [16], there was a significant increase in SUMO-2/3 modification across human genome, whereas SUMO-1 modification showed a relatively similar occupancy abet with a slight increase; these findings, suggest that KSHV specifically exploits SUMO-2/3 in order to regulate viral and host transcriptional programs.

Common binding sites that were shared by the SUMO paralogues were assessed by examining the overlap in their binding profiles between the K-Rta induced and non-induced states. The results showed that around 30% of the SUMO-1 and SUMO-2/3 binding sites under both conditions showed colocalization (Figure 1B). Interestingly, the number of SUMO-2/3 specific binding sites was increased by 12% (from ~27 k to ~57 k) while the number of SUMO-1 specific binding sites was decreased by 11% (from ~13 k to ~10 k) during viral reactivation (Figure 1B). These findings suggest that KSHV reactivation is accompanied by significant changes in the magnitude of SUMO-2/3 tagging across the genome. By contrast, the level of SUMO-1 tagging on chromatin remains relatively unchanged.

### SUMO-2/3 is enriched on the promoter regions after KSHV reactivation

**Figure 2:**
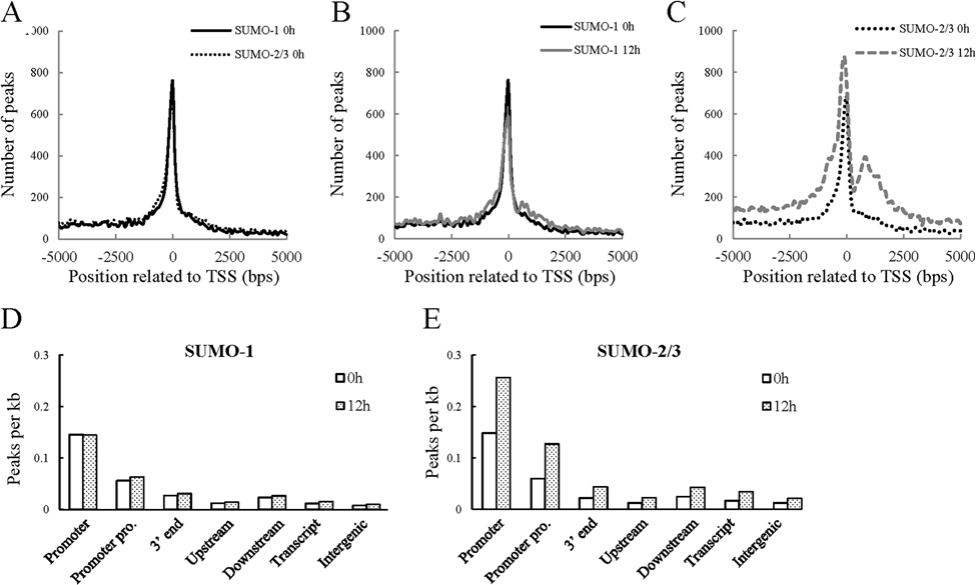
**Genome-wide analysis of SUMO-1 and SUMO-2/3 binding region during KSHV reactivation. (A-C)** Distance distribution of all SUMO-1 and SUMO-2/3 peaks. Distance to transcription start site (TSS) before **(A)** and after **(B and** **C)** K-Rta induction for viral reactivation. **(D and** **E)** Gene context of SUMO-1 **(D)** and SUMO-2/3 **(E)** binding sites during KSHV reactivation represented by peak density, after adjustment for the prevalence of gene context category in the genome. Promoter: TSS ± 500; Promoter pro. (Promoter proximal): TSS ± 2 Kb; 3′ end: TES ± 500; Upstream: -2 kb to −10 kb upstream of the TSS; Downstream: +2 kb to +10 kb downstream of the TES; Intergenic: ≥10 kb from any coding genes. TES: transcription end site.

SUMO is capable of binding to chromatin by modifying chromatin remodeling proteins and transcription factors [48]. In this context, SUMO modifications are likely to have specific distributions across the genome. As expected, SUMO target sites occur across all chromosomes but are not randomly distributed. They are enriched in regions containing genes, notably in regions annotated as promoters. As Figure 2A reveals, chromatin-bound SUMO paralogues are commonly centered and symmetrically distributed within 500 bp around transcription start sites (TSSs). This pattern is similar to that reported for chromatin modification by SUMO-1 through the cell cycle [46]. Interestingly, after overlaying the SUMO-binding data after viral reactivation onto the control SUMO-binding data, we discovered that there was a significant increase in SUMO-2/3 occupancy near to TSSs (Figure 2C) after KSHV reactivation, whereas SUMO-1 occupancy showed a slight decrease (Figure 2B). A similar pattern was identified for the groups of peaks that contain only SUMO-1 or SUMO-2/3 specific modification.

Consistent results were obtained when SUMO peaks were normalized for the size of defined genome compartment. SUMO paralogues showed a relative higher peak density in promoter regions (TSSs ± 500 bp), whereas the binding to the gene bodies themselves (transcribed regions), the transcription end sites (TESs), the regions upstream of the gene, the regions downstream of the gene and the intergenic regions were low (Figure 2D and 2E). Consistently, the peak density of SUMO-2/3 (Figure 2E), but not of SUMO-1 (Figure 2D), was significantly increased in the promoter regions during viral reactivation. These findings indicate that, while the chromatin-bound SUMO paralogues are both centered on the TSSs, only chromatin-bound SUMO-2/3 is significantly increased during KSHV reactivation.

### Global prediction of potential SUMO-1 and SUMO-2/3 targeting of chromatin-bound transcription factors

**Figure 3:**
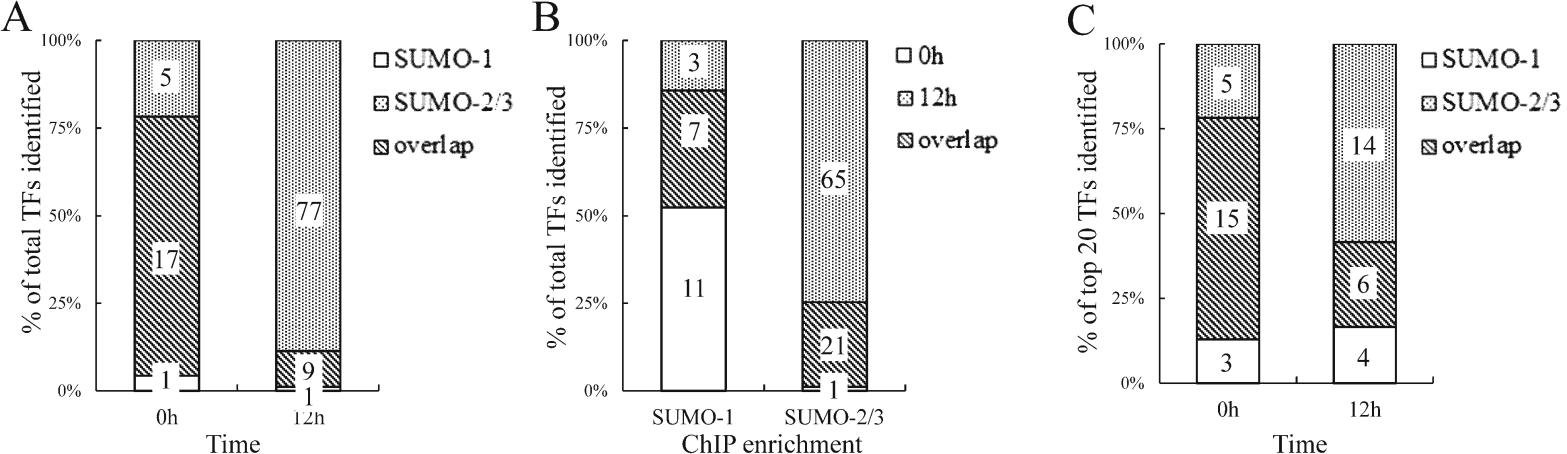
**Overview of potential TFs targeting by SUMO paralogues during KSHV reactivation. (A)** Percentage of overlapped TFs target sites for SUMO-1 and for SUMO-2/3 before and after K-Rta induction resulting in KSHV reactivation **(B)** Percentage of overlapped SUMO-1 or SUMO-2/3 target TFs before and after viral reactivation. **(C)** Percentage overlap of the top-20 potential TF targets for SUMO-1 and SUMO-2/3 before and after viral reactivation; numbers indicate overlapping and non-overlapping TF counts.

Typical SUMO binding sites are focal and consist of no more than a few hundred base pairs, a pattern reminiscent of the “peaks” associated with transcription factors. A large number of known SUMO conjugates in mammals are transcription factors. To predict the potential chromatin-associated transcription factors (TFs) that are SUMOylated, we annotate SUMO target sites within promoter regions in relation to transcription factor binding sites (TFBS) from the Transfac Matrix Database (v7.0) created by Biobase. This database contains 258 TFBS weight matrices that represent the potential DNA binding sites of 176 TFs across the genome. The SUMO enriched peaks for each TFBS in the promoter region were normalized with their own distribution frequency. Potential SUMO target TFs were ranked by percentage and Hampel Identifier was used to identify the TFBSs that were significantly correlated with SUMO binding [49]. The number of SUMO peaks identified before viral reactivation was used as the control. High confidence TFBSs that correlated with SUMO-1 and SUMO-2/3 peaks were mapped and are represented here as “potential SUMO-1 and SUMO-2/3 target TFs”. Interestingly, during viral reactivation, the SUMO-1 target TFs decreased from 18 to 10, while the SUMO-2/3 target TFs were significantly increased from 22 to 86 (Figure 3A). When we overlapped the potential SUMO-1 and SUMO-2/3 target TFs, we found that 74% of the TFs shared by SUMO paralogues in the non-reactivated control cells and this decreased (10%) during viral reactivation (Figure 3A). Around 20 ~ 30% of the potential SUMO-1 and SUMO-2/3 target TFs overlapped before and after KSHV reactivation (Figure 3B). Moreover, SUMO-1 target TFs consisted of more non-overlapping TFs before viral reactivation (11 of 21), while, on the other hand, there were more non-overlapping TFs for SUMO-2/3 (65 of 87) that were recognized after viral reactivation (Figure 3B). Collectively, these results suggest that SUMO-2/3 significantly increased its tagging of TFs bound to promoter regions during KSHV reactivation.

The top twenty potential SUMO-1 and SUMO-2/3 target TFs before and after KSHV reactivation are listed in Tables 1, 2, 3 and 4. If less than twenty TFs have been identified, all of them are listed. One interesting point involves the reasons for SUMO-2/3 target TFs identification after viral reactivation, which is quite different from that for SUMO-1 target TFs. As shown in Table 4, there is a more significant increase in peak numbers for the top-20 SUMO-2/3 target TFs after KSHV reactivation comparing with the top twenty TFs listed from the non-reactivated control cells (Table 2). In contrast, the peak number for the SUMO-1 target TFs after viral reactivation almost all decreased (Table 3); this decrease is less than that of the control (Table 1). The findings indicate an increase in SUMO-2/3 modification of TFs during viral reactivation. In contrast, SUMO-1 modification of TFs after viral reactivation had decreased. These finding suggest that SUMO paralogues are differentially regulated in a global manner under certain circumstances, for example when there is viral reactivation as is the case here. The SUMO-1 and SUMO-2/3 specific TFBSs identified here provides a framework that allows the study of the potential functional differences between SUMO paralogues.

**Table 1:** **Top 20 potential SUMO-1 targeting TFs before KSHV reactivation**

Rank	Name	Transfac	TFBS # (in TSS±500 bp)	Peak # of TFBS (in TSS±500 bp)		% of TFBS Peak (in TSS±500 bp)		Gene #	
				0 h	12 h	0 h	12 h	0 h	12 h
1	**ETS1**	V$CETS1P54_01	1587	341	262	21.5%	16.5%	294	229
2	GABPA/NRF-2	V$NRF2_01	1577	336	237	21.3%	15.0%	286	206
3	**ELK1**	V$ELK1_02	1667	333	254	20.0%	15.2%	279	215
4	USF1	V$USF_C	441	79	45	17.9%	10.2%	71	44
		V$USF_Q6	1136	146	97	12.9%	8.5%	132	92
5	**STAT1**	V$STAT1_03	282	50	41	17.7%	14.5%	48	39
		V$STAT1_01	1179	146	122	12.4%	10.3%	134	110
6	STAT4	V$STAT4_01	106	17	17	16.0%	16.0%	4	4
7	**MYC**/MAX	V$MYCMAX_02	580	90	76	15.5%	13.1%	68	55
		V$MYCMAX_01	784	104	88	13.3%	11.2%	93	68
8	**CREB1**	V$CREB_01	666	103	87	15.5%	13.1%	97	83
9	MAX	V$MAX_01	584	87	68	14.9%	11.6%	75	64
10	ATF-2	V$CREBP1_01	272	37	30	13.6%	11.0%	35	28
11	**STAT3**	V$STAT3_01	986	133	108	13.5%	11.0%	123	101
12	**SREBF1/SREBP1**	V$SREBP1_01	1038	140	108	13.5%	10.4%	110	93
13	**MYCN**	V$NMYC_01	1467	195	125	13.3%	8.5%	170	114
14	STAT6	V$STAT_01	570	75	59	13.2%	10.4%	66	54
15	**ARNT**	V$ARNT_01	835	108	89	12.9%	10.7%	102	87
16	ARID5B/MRF2	V$MRF2_01	478	61	57	12.8%	11.9%	56	52
17	**E2F1**/2/3/4/5	V$E2F_02	1301	166	121	12.8%	9.3%	149	110
18	PBX1	V$PBX1_01	220	28	21	12.7%	9.5%	28	21

bold: transcription factor with evidence of SUMO modification.

**Table 2:** **Top 20 potential SUMO-2/3 targeting TFs before KSHV reactivation**

Rank	Name	Transfac	TFBS # (in TSS±500 bp)	Peak # of TFBS (in TSS±500 bp)		% of TFBS Peak (in TSS±500 bp)		Gene #	
				0 h	12 h	0 h	12 h	0 h	12 h
1	**ETS1**	V$CETS1P54_01	1587	337	397	21.2%	25.0%	296	353
2	GABPA/NRF-2	V$NRF2_01	1577	320	379	20.3%	24.0%	274	335
3	**ELK1**	V$ELK1_02	1667	336	382	20.2%	22.9%	285	328
4	STAT4	V$STAT4_01	106	20	12	18.9%	11.3%	7	12
5	**STAT1**	V$STAT1_03	282	53	79	18.8%	28.0%	45	68
6	**CREB1**	V$CREB_01	666	124	164	18.6%	24.6%	114	152
7	ATF2	V$CREBP1_01	272	45	67	16.5%	24.6%	45	64
		V$CREBP1_Q2	1273	168	201	13.2%	15.8%	135	198
8	STAT6	V$STAT_01	570	92	122	16.1%	21.4%	85	110
9	**MYC**/MAX	V$MYCMAX_02	580	93	96	16.0%	16.6%	74	87
10	MAX	V$MAX_01	584	93	110	15.9%	18.8%	79	97
11	HLF	V$HLF_01	438	69	90	15.8%	20.5%	66	85
12	USF1	V$USF_C	441	69	86	15.6%	19.5%	64	78
13	ARID5B/MRF2	V$MRF2_01	478	74	100	15.5%	20.9%	68	91
14	NFYA/B/C	V$NFY_Q6	1557	217	295	13.9%	18.9%	176	250
		V$NFY_C	416	57	79	13.7%	19.0%	55	75
15	**SREBF1/SREBP1**	V$SREBP1_01	1038	144	179	13.9%	17.2%	100	170
16	**STAT5A**	V$STAT5A_02	982	135	155	13.7%	15.8%	109	133
17	ZEB1	V$AREB6_04	530	70	89	13.2%	16.8%	60	78
18	**ARNT**	V$ARNT_01	835	110	136	13.2%	16.3%	106	131
19	**E2F1**/2/3/4/5	V$E2F_02	1301	171	226	13.1%	17.4%	155	199
20	**STAT3**	V$STAT3_01	986	129	168	13.1%	17.0%	127	159

bold: transcription factor with evidence of SUMO modification.

**Table 3:** **Top 20 potential SUMO-1 targeting TFs after KSHV reactivation**

Rank	Name	Transfac	TFBS # (in TSS±500 bp)	Peak # of TFBS (in TSS±500 bp)		% of TFBS Peak (in TSS±500 bp)		Gene #	
				0 h	12 h	0 h	12 h	0 h	12 h
1	**ETS1**	V$CETS1P54_01	1587	341	262	21.5%	16.5%	294	229
2	STAT4	V$STAT4_01	106	17	17	16.0%	16.0%	4	4
3	**ELK1**	V$ELK1_02	1667	333	254	20.0%	15.2%	279	215
4	GABPA/NRF-2	V$NRF2_01	1577	336	237	21.3%	15.0%	286	206
5	**STAT1**	V$STAT1_03	282	50	41	17.7%	14.5%	48	39
6	**NFATC1**/2/3/4	V$NFAT_Q6	487	48	70	9.9%	14.4%	22	23
7	**STAT5A**	V$STAT5A_02	982	118	129	12.0%	13.1%	95	104
8	**MYC**/MAX	V$MYCMAX_02	580	90	76	15.5%	13.1%	68	55
9	**CREB1**	V$CREB_01	666	103	87	15.5%	13.1%	97	83
10	CUTL1	V$CDPCR1_01	582	59	73	10.1%	12.5%	54	49

bold: transcription factor with evidence of SUMO modification.

**Table 4:** **Top 20 potential SUMO-2/3 targeting TFs after KSHV reactivation**

Rank	Name	Transfac	TFBS # (in TSS±500 bp)	Peak # of TFBS (in TSS±500 bp)		% of TFBS Peak (in TSS±500 bp)		Gene #	
				0 h	12 h	0 h	12 h	0 h	12 h
1	**STAT1**	V$STAT1_03	282	53	79	18.8%	28.0%	45	68
2	**ETS1**	V$CETS1P54_01	1587	337	397	21.2%	25.0%	296	353
3	PBX1	V$PBX1_01	220	28	55	12.7%	25.0%	28	54
4	ATF2	V$CREBP1_01	272	45	67	16.5%	24.6%	45	64
5	**CREB1**	V$CREB_01	666	124	164	18.6%	24.6%	114	152
6	GABPA/NRF-2	V$NRF2_01	1577	320	379	20.3%	24.0%	274	335
7	**ELK1**	V$ELK1_02	1667	336	382	20.2%	22.9%	285	328
8	**IRF-1**	V$IRF1_01	534	46	118	8.6%	22.1%	43	113
9	STAT6	V$STAT_01	570	92	122	16.1%	21.4%	85	110
10	FOXO4	V$FOXO4_02	456	28	97	6.1%	21.3%	28	75
11	**NFATC1**/2/3/4	V$NFAT_Q6	487	50	102	10.3%	20.9%	29	55
12	ARID5B/MRF2	V$MRF2_01	478	74	100	15.5%	20.9%	68	91
13	**IRF-2**	V$IRF2_01	601	48	125	8.0%	20.8%	63	93
14	SRY	V$SRY_01	523	32	108	6.1%	20.7%	30	91
15	HLF	V$HLF_01	438	69	90	15.8%	20.5%	66	85
16	**IRF-7**	V$IRF7_01	754	87	153	11.5%	20.3%	79	126
17	**MEF2A**	V$MEF2_01	455	36	91	7.9%	20.0%	28	72
18	**GATA1**	V$GATA1_04	191	14	38	7.3%	19.9%	13	35
19	POU3F2	V$POU3F2_02	246	17	48	6.9%	19.5%	17	43
20	USF1	V$USF_C	441	69	86	15.6%	19.5%	64	78

bold: transcription factor with evidence of SUMO modification.

### Identification of potential transcription factors targeted by SUMO-2/3 during KSHV reactivation

**Figure 4:**
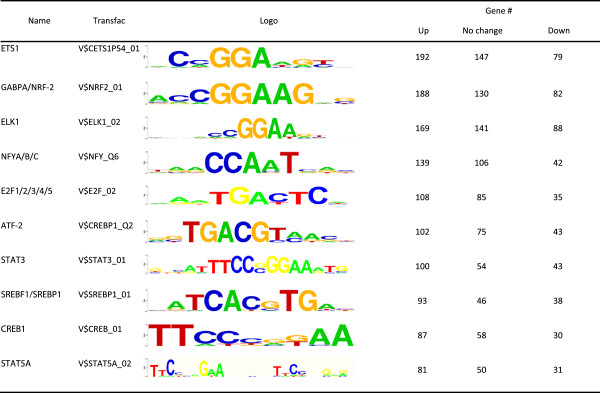
**Top 10 potential SUMO-2/3 targeting TFs ranking by gene number before KSHV reactivation.** bold: transcription factor with evidence of SUMO modification.

**Figure 5:**
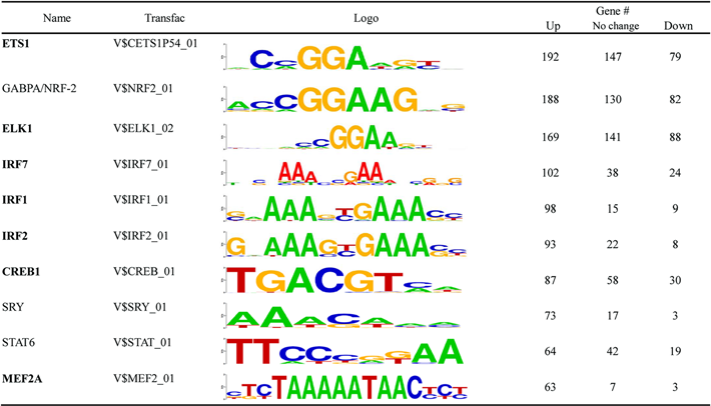
**Top 10 potential SUMO-2/3 targeting TFs ranking by gene number after KSHV reactivation.** bold: transcription factor with evidence of SUMO modification.

**Figure 6:**
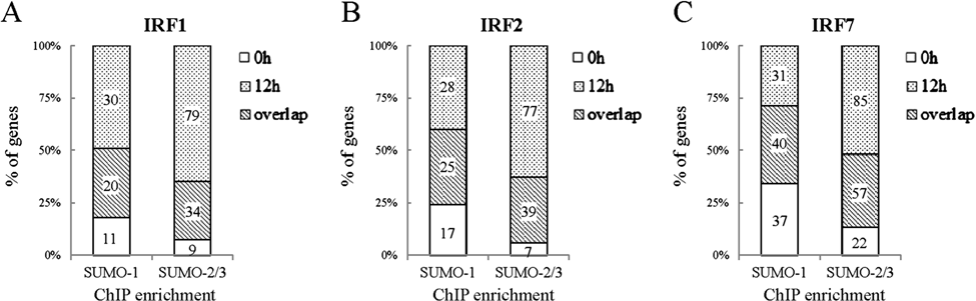
**Overview of SUMO enrichment at IRF-1, IRF-2 and IRF-7 binding sites during viral reactivation.** Percentage of SUMO-1 and SUMO-2/3 target gene numbers with SUMO enrichment at IRF-1 **(A)**, IRF-2 **(B)** or IRF-7 **(C)** binding sites in promoter regions before and after K-Rta induction for KSHV reactivation; numbers indicate counts of overlapping and non-overlapping gene numbers.

**Figure 7:**
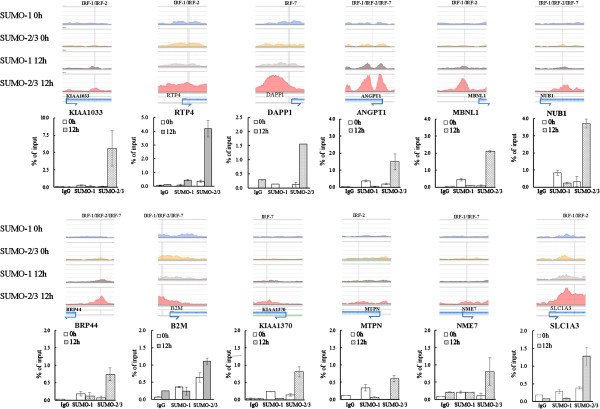
**Confirmation of data derived from ChIP-seq for IRF-1, IRF-2 and IRF-7 binding sites with SUMO-2/3 enrichment relevant to K-Rta induction of KSHV reactivation in BCBL-1 cells.** Chromatin samples derived from K-Rta-inducible BCBL-1 cells before and after 12 hours of K-Rta induction were used in ChIP reactions with antibodies specific for SUMO-1 and SUMO-2/3. Following ChIP assay, the IRF binding sites within the promoters of the genes, which are indicated at the bottom of the figure, were amplified using qPCR. All reactions were run in triplicate and normalized against the input. Nonspecific IgG was used as the control ChIP antibody.

**Figure 8:**
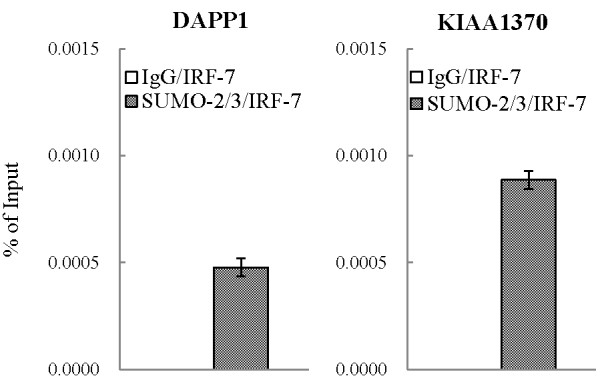
**IRF-7 and SUMO-2/3 co-localized at IRF-7 binding sites with SUMO-2/3 binding enrichment after KSHV reactivation.** Quantification of DNA recovered from DAPP1 and KIAA1370 promoters by real-time qPCR after enrichment by ChIP with rabbit non-immune serum IgG or anti-SUMO-2/3 antibodies and reChIP of IRF-7 with anti-IRF-7 antibody. One non-IRF-7 target gene, KIAA1033, was used in qPCR as negative controls.

The presence of highly enriched SUMO-2/3 binding sites around the promoter regions during viral reactivation suggest that SUMO-2/3 might be directly or indirectly targeting a large group of transcription factors during KSHV reactivation. In order to pinpoint the most important gene-regulating TFs that are targeted by SUMO-2/3 during viral reactivation, we collected genes with SUMO-2/3 targeted TFBSs at their promoter before and after viral reactivation and group them into an up-group (SUMO peaks increase >1.5X), a down-group (SUMO peaks decrease >1.5X) and a no-change-group (SUMO peaks variants within 1.5X). When we ranked the TFs by total gene number, the top 10 most important gene-regulating TFs targeted by SUMO-2/3 after KSHV reactivation could be identified (Figures 4 and 5). Interestingly, we found that there were three IRFs, IRF-7, IRF-1, and IRF-2 that do not exist in the SUMO-2/3 target TFs list before viral reactivation, but are now listed as the 4th, 5th and 6th top-most TFs, respectively, after viral reactivation. Comparing the SUMO tagging of these IRFs before and after viral reactivation, we found that all three IRFs binding sites are preferentially subjected to SUMO-2/3 modification after viral reactivation (Figure 6). To confirm that SUMO-2/3 enrichment at the IRF-1, IRF-2 and IRF-7 binding sites occurs during viral reactivation, we design primers targeting the IRFs binding regions where the SUMO-2/3 peaks had been identified by the ChIP-seq assay. The SUMO-2/3 enrichment in those regions was then validated using a ChIP sample and real-time quantitative-PCR (qPCR). Consistent with the ChIP-seq results, the 12 IRF binding regions tested here showed significant enrichment after viral reactivation for SUMO-2/3 but not for SUMO-1 compare to the non-reactivated control cells (Figure 7). ChIP-reChIP analyses further confirmed the co-localization of IRF-7 and SUMO-2/3 on IRF-7 binding region with SUMO-2/3 enrichment (Figure 8).

**Figure 9:**
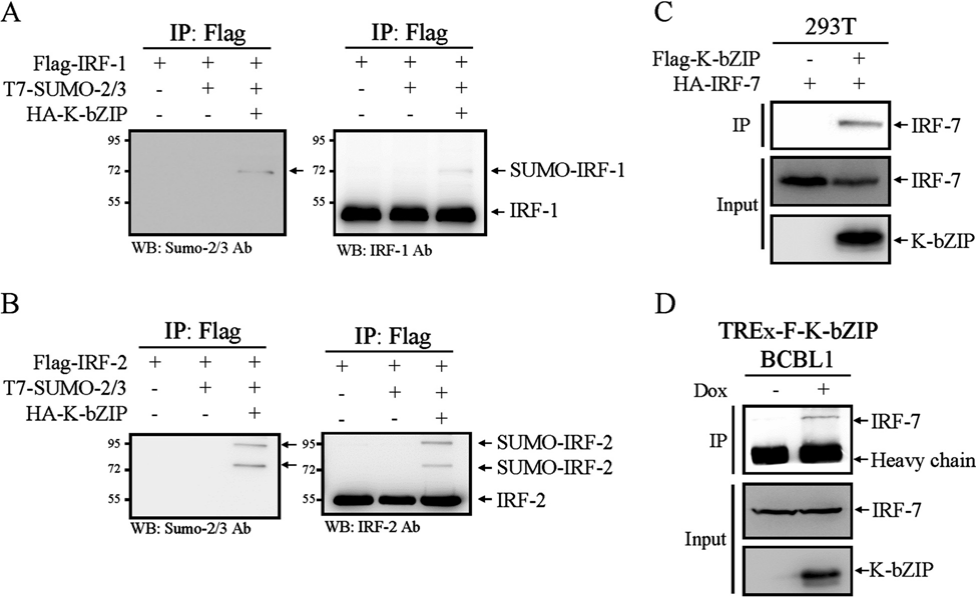
**K-bZIP is able to SUMOylate IRF-1 and IRF-2 and to interact with IRF-7.** Flag-tagged IRF-1 **(A)**, IRF-2 **(B)** and IRF-7 **(C)** were transiently co-transfected with the indicated tagged constructs. Total cell lysates (TCLs) were prepared 48 hours after transfection. The lysates were immunoprecipitated with M2-beads and analyzed by immunoblotting using anti-IRF-1 (**A**; right panel), anti-IRF-2 (**B**; right panel), anti-SUMO-2/3 (**A** and **B**; left panel) and anti-IRF-7 **(C)** antibodies. **(D)** TCLs from TREx-F3H3-K-bZIP BCBL-1 cells before and after 48 hours of Dox induction for K-bZIP overexpression were used for immunoprecipitation with anti-K-bZIP antibody. The immunoprecipitates and TCLs were analyzed by immunoblot with antibodies as indicated.

The identification of IRF-1, IRF-2 and IRF-7 as potential SUMO-2/3 targets during KSHV reactivation suggests that the viral SUMO E3 ligase K-bZIP may be involved in this phenomenon. To address this, we first cloned all three IRFs using cDNA of BCBL-1 cells. Then 293 T cells were transiently co-transfected with Flag-tagged IRF-1, IRF-2 or IRF-7, T7-tagged SUMO-2 and SUMO-3, and HA-tagged K-bZIP; this was followed by immunoblotting or immunoprecipitation using Flag antibody. The results showed an increase in SUMO modification of IRF-1 and IRF-2 when there was overexpression of K-bZIP (Figure right panel of 9A and B). SUMOylation of IRF-1 and IRF-2 was further confirmed by immunoblotting using anti-SUMO-2/3 antibody (Figure left panel of 9A and B). Although we were unable to identify SUMOylation of IRF-7 using this approach (data not shown), an immunoprecipitation assay showed that IRF-7 is able to interact with K-bZIP (Figure 9C and D). Thus SUMO-mediated transcription regulation not only involves covalent SUMO modification of transcription regulatory proteins, but also seems to involve SUMO modified co-regulatory proteins that show a non-covalent association at the TFBS. These findings suggest that IRF-7 may recruit K-bZIP to its binding sites together with other K-bZIP SUMOylated chromatin binding protein(s); these are then able to be co-immunoprecipitated (co-IPed) by SUMO antibody.

### SUMO-2/3 is enriched on promoters of immune-related genes that are unaltered during KSHV reactivation

**Figure 10:**
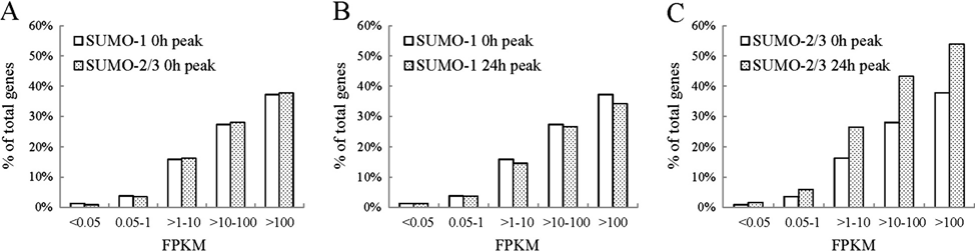
**SUMO-2/3 are recruited to the promoters of genes with medium-level and high-level expression after KSHV reactivation. (A)** Occupancy with SUMO-1 and SUMO-2/3 at the promoters of genes were categorized in terms of gene expression level from low to high in the control cells. (**B** and **C**) Occupancy of SUMO-1 **(B)** and SUMO-2/3 **(C)** after KSHV reactivation was plotted in a similar manner to **(A)** and compared with the control cells.

To study the functional role of SUMO-2/3 in the regulation of gene expression during KSHV reactivation, we conducted a detailed RNA-seq analysis using TREx-F3H3-K-Rta BCBL-1 cells before and after K-Rta induction for viral reactivation. We sorted 26008 genes using expression levels based on FPKM into five groups, namely no expression (FPKM <0.05: 7954), low expression (FPKM 0.05 ~ <1: 6503), medium expression (FPKM 1 ~ <10: 5597), high expression (FPKM 10 ~ <100: 5390), and very high expression (FPKM >100: 564). We found that between 27% and 37% of the very high and high expression group, about 16% of the medium expression group, about 4% of the low expression group, and about 1% of the no expression group promoters were labeled by SUMO-1 or SUMO-2/3 (Figure 10A). Consistent with a previous study using yeast [7] and a study of SUMO-1 using HeLa cells [46], both of which showed that SUMO preferentially occupies transcriptionally active genes, the modification by all SUMO paralogues explored here also seems to be present at greater levels on the promoters of genes that show a higher level of expression. Twenty-four hours after KSHV reactivation, we found a significant increase in SUMO-2/3 binding at the promoters of genes with high expression (~15%) and medium expression (~10%). This compared with little SUMO-2/3 binding enrichment at the promoters of genes with low expression (~2%) and no expression (<1%) (Figure 10C). In contrast, there was a slight decrease in SUMO-1 modification across all expression categories (Figure 10B). These results indicate that SUMO-1 and SUMO-2/3 modifications are important for maintaining the transcription profiles of the non-reactivated control cells. Nevertheless, the increase in SUMO-2/3 modification during KSHV reactivation supports the notion that specific SUMO-2/3 targeting is important to transcription regulation during the KSHV life cycle.

**Figure 11:**
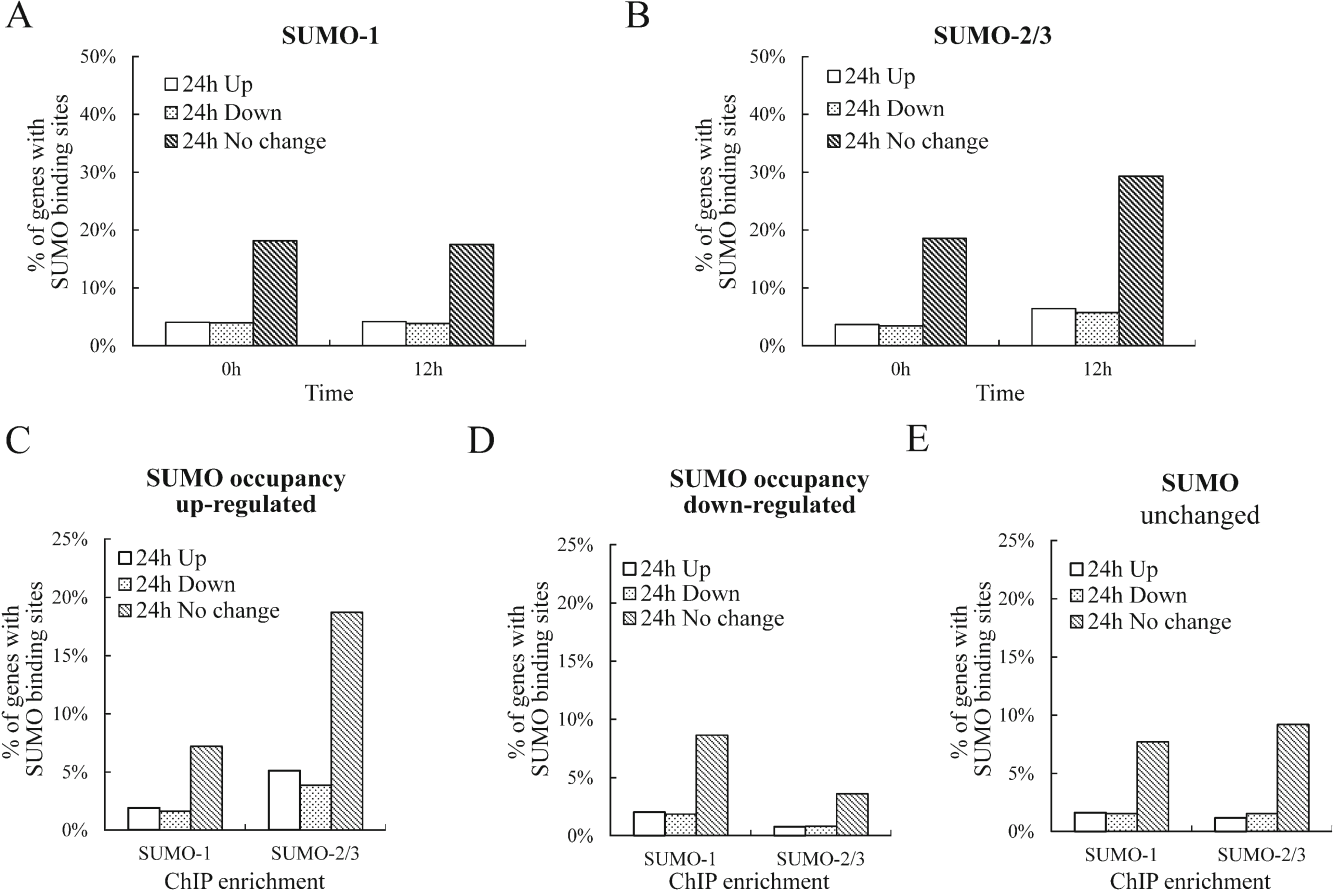
**The distribution of transcriptional up-regulated, down-regulated and unchanged genes has SUMO binding within their promoter region before and after viral reactivation.** **(A and** **B)** Genes transcriptionally up-regulated, down-regulated and unchanged after viral reactivation were plotted against SUMO-1 **(A)** or SUMO-2/3 **(B)** occupancy on the promoter region of control (0 hr; left panel) and KSHV reactivated (12 hr; right panel) BCBL-1 cells. **(C to** **E)** Genes transcriptionally up-regulated, down-regulated and unchanged after viral reactivation were plotted against SUMO-1 (left panel) and SUMO-2/3 (right panel) occupancy that was increased **(C)**, decreased **(D)** or unchanged **(E)** on the promoter region during KSHV reactivation. Bars represent % of transcriptionally up-regulated, down-regulated and unchanged genes with SUMO occupancy that was normalized to total transcriptionally up-regulated, down-regulated and unchanged genes during viral reactivation, respectively.

**Figure 12:**
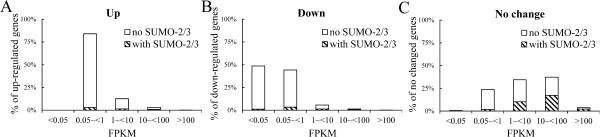
**The association of the transcription level and the up-regulated, down-regulated and no change genes during KSHV reactivation.** Genes in the up-regulated **(A)**, down-regulated **(B)** and no-change **(C)** sets were categorized by gene expression level from low to high. Percentages of SUMO-2/3 occupancy at the promoters of genes with different transcription levels were plotted as slanted lines.

**Figure 13:**
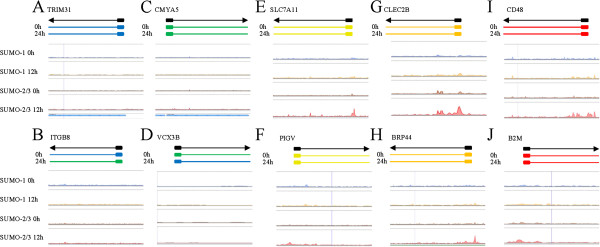
**Histogram of SUMO paralogue binding sites before and after KSHV reactivation.** Examples of the epigenetic features associated with no expression **(A and** **B)**, low expression **(C and** **D)**, medium expression **(E and** **F)**, high expression **(G and** **H)**, and very high expression **(I and** **J)** gene loci. No expression: blue; low expression: green; medium expression: yellow; high expression: orange; very high expression: red.

**Figure 14:**
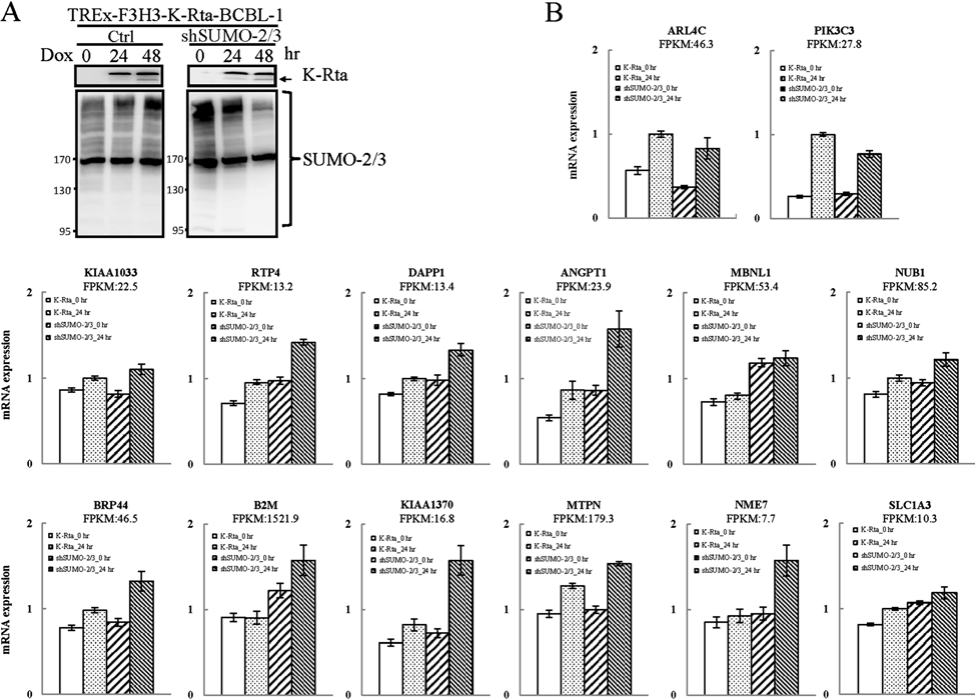
**SUMO-2/3 enrichment in stabilizing gene transcriptional during KSHV reactivation. (A)** TREx-F3H3-K-Rta-shSUMO-2/3 BCBL-1 cells were treated with Dox for 24 and 48 hours. TCLs were analyzed by immunoblotting using anti-SUMO-2/3 antibody. **(B)** Twelve IRF-1, IRF-2 and IRF-7 targeted genes showing SUMO-2/3 enrichment at the promoter region during KSHV reactivation were chosen. Two genes showing no SUMO-2/3 enrichment at the promoter region were chosen as control. RNA samples derived from TREx-F3H3-K-Rta BCBL-1 and TREx-F3H3-K-Rta-shSUMO-2/3 BCBL-1 cells before and after 24 hours of Dox induction were subjected to reverse transcription (RT) reaction. Following the RT reaction, the IRF target genes were amplified by qPCR using gene-specific primer sets. All reactions were run in triplicate and normalized against GAPDH.

In addition to maintaining constitutive transcription, SUMO has also been found to prevents the overt activation of induced genes by facilitating the shut off of the transcription in yeast [7]. To assess the effect of SUMO-2/3 enrichment on the shut-off of transcription, we compared global host gene expression in BCBL-1 cells before and after KSHV reactivation. We found that among the ~18,000 transcriptionally active host genes (genes with FPKM >0.05), only ~2,600 of the up-regulated genes and ~2,200 of the down-regulated genes were changed more than 1.5-fold in response to KSHV reactivation for 24 hours. A similar result was found at 12 hours after viral reactivation (~1,900 up-regulated and ~2,100 down-regulated genes). Analysis of the SUMO peak distributions within the promoter region of these transcriptionally up-regulated, down-regulated, and no change genes showed that the predominant association is between SUMO enrichment peaks and genes that can be shown to exhibit no change in expression. After viral reactivation for 24 hours, there was a significant increase in SUMO-2/3 recruitment to the promoters of transcriptionally unaltered genes (Figure 11A and B). When we further grouped the SUMO peaks into increased binding, decrease binding and no change in binding during viral reactivation, a similar result was found (Figure 11C to E). SUMO-2/3 peak enrichment during viral reactivation was predominantly associated with transcriptionally unaltered genes. When we analyzed the association of the expression level of the transcriptionally up-regulated, down-regulated and unaltered genes with SUMO peaks during viral reactivation, we found that most of the viral up-regulated (>80%) and down-regulated (>90%) genes fall into the low (FPKM 0.05 ~ 1) and no expression (FPKM <0.05) gene categories that show little SUMO-2/3 modification on their promoter (Figure 12A and B). Furthermore and interestingly, more than 75% of the expression-unchanged genes, which contain a higher proportion of SUMO-2/3 modification on their promoter, are in the medium and high expression gene categories (Figure 12C and Figure 13). This result indicates that SUMO-2/3 enrichment during viral reactivation may contribute to “stabilizing” the transcriptional activity of these medium (FPKM 1 ~ 10) and high (FPKM >10) expression genes during viral reactivation. To confirm the RNA-seq data, we design primers for IRF-1, IRF-2 and IRF-7 targeted and transcriptionally active genes that show no change in expression during viral reactivation in BCBL-1 cells. The lack of change in expression during viral reactivation was confirmed using cDNA samples and real-time qPCR. Consistent with the RNA-seq results, the 12 genes tested here showed no changes in expression level compared to the control after K-Rta-induced KSHV reactivation (Figure 14). To further study the “stabilizing” potential of SUMO-2/3 in transcription regulation, we generated an inducible SUMO-2/3 knockdown BCBL-1 cell line, TREx-F3H3-K-Rta-shSUMO-2/3 BCBL-1. Western blot analysis shows the successful knockdown of SUMO-2/3 at 24 and 48 hours after induction (Figure 14A). Consistent with our hypothesis, qPCR analysis showed a higher induction of most of the 12 genes we analyzed after SUMO-2/3 knockdown during viral reactivation (Figure 14B). Again, these results imply that SUMO-2/3 enrichment within the host promoter region during KSHV reactivation is closely related to preventing transcriptional activation of constitutively active host genes, many of which are immune response genes (see below).

To determine whether SUMO-2/3 target a group of genes with specific functions, we carried out a gene ontology (GO) analysis of genes that are targeted by SUMO-1 and SUMO-2/3 before and after viral reactivation using the IPA software. We found that the genes targeted by SUMO-2/3 after viral reactivation are significantly involved in several pathways related to cellular immune responses, cytokine signaling, cell growth, apoptosis and cancer (Table 5). We further analyzed SUMO targeted genes with no change in transcription and genes with no change in transcription but increased in SUMO-2/3 enrichment. Consistently, we found that the transcriptionally unaltered genes targeted by SUMO-2/3 after viral reactivation are significantly involved in cellular immune responses (Table 6). Taken together, all of the present results support the notion that KSHV may target SUMO-2/3 modified proteins to active chromatin regions to prevent overt activation of various important genes during viral reactivation, especially those involved in the innate immune response.

**Table 5:** **GO categories of SUMO targeting genes during KSHV reactivation**

			SUMO-1				SUMO-2/3			
			0 h		12 h		0 h		12 h	
	Pathway category	Genes in category	Genes identified	***p***-value	Genes identified	***p***-value	Genes identified	***p***-value	Genes identified	***p***-value
**Pathogen-influenced signaling**	Virus entry via endocytic pathways	99	14	2.59E-01	16	8.71E-02	13	3.35E-01	31	**1.41E-03**
**Cellular immune response**	Antigen presentation pathway	40	8	9.77E-02	5	5.02E-01	8	8.71E-02	14	**1.26E-02**
	CD40 signaling	70	11	1.87E-01	12	8.91E-02	11	1.67E-01	23	**2.82E-03**
	HMGB1 signaling	99	15	2.19E-01	18	**3.89E-02**	13	3.96E-01	25	9.33E-02
	IL-10 signaling	78	13	9.55E-02	14	**4.07E-02**	10	3.58E-01	19	9.55E-02
	IL-15 signaling	67	8	5.43E-01	12	7.41E-02	8	5.15E-01	20	**1.86E-02**
	IL-3 signaling	74	11	3.11E-01	12	1.74E-01	10	4.11E-01	23	**1.41E-02**
	IL-8 signaling	205	29	1.52E-01	28	1.61E-01	29	1.25E-01	48	**4.68E-02**
	NF-κB activation by viruses	82	−	−	12	1.99E-01	−	−	23	**2.00E-02**
	p38 MAPK signaling	117	22	**4.47E-02**	18	2.10E-01	17	3.10E-01	26	3.80E-01
	Toll-like receptor signaling	62	14	**1.05E-02**	9	2.65E-01	−	−	17	5.75E-02
**Cytokine signaling**	IL-17A signaling in airway cells	72	9	4.33E-01	12	8.91E-02	10	2.72E-01	21	**1.35E-02**
	Prolactin signaling	80	10	4.78E-01	13	1.21E-01	−	−	22	**3.72E-02**
**Cancer**	Glioma signaling	112	17	1.03E-01	14	3.01E-01	−	−	29	**1.55E-02**
	p53 signaling	96	18	5.13E-02	16	1.17E-01	16	1.24E-01	32	**1.48E-03**
	Small cell lung cancer signaling	89	13	1.24E-01	13	1.02E-01	13	1.08E-01	27	**6.03E-04**
	Sonic hedgehog signaling	33	6	1.65E-01	9	**7.24E-03**	5	3.03E-01	−	−
**Cellular growth, proliferation and development**	Cleavage and polyadenylation of Pre-mRNA	12	5	**1.32E-02**	4	5.37E-02	3	1.87E-01	5	8.32E-02
	EGF signaling	62	11	1.02E-01	8	4.05E-01	−	−	21	**2.88E-03**
	FAK signaling	101	−	−	15	1.15E-01	12	4.06E-01	27	**1.32E-02**
	JAK/Stat signaling	70	13	8.71E-02	13	7.08E-02	10	3.40E-01	26	**5.37E-04**
	Oncostatin M signaling	35	5	4.55E-01	5	4.26E-01	−	−	12	**3.55E-02**
	PDGF signaling	85	12	2.92E-01	12	2.54E-01	−	−	25	**1.07E-02**
	Thrombopoietin signaling	63	8	4.22E-01	10	1.45E-01	−	−	17	**4.90E-02**
	VEGF signaling	103	17	7.59E-02	19	**1.66E-02**	13	3.66E-01	25	7.59E-02
**Cell cycle regulation**	Cyclins and cell cycle regulation	89	14	1.38E-01	11	4.05E-01	15	6.92E-02	24	**2.75E-02**
	GADD45 signaling	22	4	2.26E-01	3	4.33E-01	5	8.32E-02	9	**8.51E-03**
	Mitotic roles of polo-like kinase	70	11	2.46E-01	11	2.14E-01	14	**3.89E-02**	18	1.39E-01
**Growth factor signaling**	ErbB2-ErbB3 signaling	60	−	−	11	9.55E-02	−	−	19	**1.82E-02**
**Apoptosis**	April mediated signaling	43	8	1.10E-01	6	3.40E-01	8	1.00E-01	13	**3.72E-02**
	Aryl hydrocarbon receptor signaling	161	24	8.51E-02	19	3.81E-01	23	1.07E-01	40	**1.48E-02**
	Myc mediated apoptosis signaling	60	10	2.13E-01	10	1.85E-01	9	3.10E-01	20	**1.05E-02**
**Cellular stress and injury**	Endoplasmic reticulum stress pathway	18	7	**5.25E-03**	5	6.61E-02	5	6.76E-02	7	6.03E-02
**Disease-specific pathways**	Parkinson’s signaling	16	3	3.44E-01	4	1.31E-01	5	**4.27E-02**	4	4.32E-01
**Neurotransmitters and other nervous system signaling**	Cholecystokinin/gastrin-mediated signaling	106	21	**1.82E-02**	13	5.05E-01	15	2.94E-01	24	2.59E-01

bold: with statistical significance.

**Table 6:** **GO categories of SUMO targeting genes with no expression changes during KSHV reactivation**

			SUMO-2/3 12 h				SUMO-2/3 enriched at 12 hr	
			All genes		Gene expression no change		Gene expression no change	
	Pathway category	Genes in category	Genes identified	***p***-value	Genes identified	***p***-value	Genes identified	***p***-value
**Pathogen-influenced signaling**	Virus entry via endocytic pathways	99	31	**1.41E-03**	28	**3.24E-03**	20	**5.13E-03**
**Cellular immune response**	Antigen Presentation pathway	40	14	**1.26E-02**	12	**3.63E-02**	9	**3.24E-02**
	CD40 signaling	70	23	**2.82E-03**	21	**4.79E-03**	14	**1.91E-02**
	HMGB1 signaling	99	25	9.33E-02	24	6.46E-02	16	1.03E-01
	IL-10 signaling	78	19	9.55E-02	16	2.07E-01	11	2.10E-01
	IL-15 signaling	67	20	**1.86E-02**	19	**1.55E-02**	16	**2.34E-03**
	IL-3 signaling	74	23	**1.41E-02**	22	**1.02E-02**	14	**4.90E-02**
	IL-8 signaling	205	48	**4.68E-02**	44	6.92E-02	27	2.14E-01
	NF-κB activation by viruses	82	23	**2.00E-02**	21	**3.16E-02**	15	**3.39E-02**
	p38 MAPK signaling	117	26	3.80E-01	24	3.88E-01	16	3.79E-01
	Toll-like receptor signaling	62	17	5.75E-02	15	9.55E-02	11	7.76E-02
**Cytokine signaling**	IL-17A signaling in airway cells	72	21	**1.35E-02**	2	8.51E-02	11	1.58E-01
	Prolactin signaling	80	22	**3.72E-02**	22	**1.45E-02**	17	**6.46E-03**
**Cancer**	Glioma signaling	112	29	**1.55E-02**	27	**1.62E-02**	19	**2.14E-02**
	p53 signaling	96	32	**1.48E-03**	27	**1.23E-02**	20	**8.51E-03**
	Small cell lung cancer signaling	89	27	**6.03E-04**	26	**3.63E-04**	20	**2.57E-04**
**Cellular growth, proliferation and development**	Cleavage and polyadenylation of pre-mRNA	12	5	8.32E-02	5	6.03E-02	4	5.01E-02
	EGF signaling	62	21	**2.88E-03**	20	**2.34E-03**	13	**1.66E-02**
	FAK signaling	101	27	**1.32E-02**	26	**9.33E-03**	17	**3.47E-02**
	JAK/Stat signaling	70	26	**5.37E-04**	24	**8.71E-04**	20	**1.07E-04**
	Oncostatin M signaling	35	12	**3.55E-02**	11	**4.47E-02**	8	5.01E-02
	PDGF signaling	85	25	**1.07E-02**	24	**6.92E-03**	18	5.01E-03
	Thrombopoietin signaling	63	17	**4.90E-02**	17	**2.29E-02**	12	**3.31E-02**
	VEGF signaling	103	25	7.59E-02	25	**3.47E-02**	16	9.55E-02
**Cell cycle regulation**	Cyclins and cell cycle regulation	89	24	**2.75E-02**	23	**2.19E-02**	13	1.80E-01
	GADD45 signaling	22	9	**8.51E-03**	8	**2.40E-02**	4	2.26E-01
	Mitotic roles of polo-like kinase	70	18	1.39E-01	18	7.24E-02	9	4.44E-01
**Growth Ffactor signaling**	ErbB2-ErbB3 signaling	60	19	**1.82E-02**	19	**7.08E-03**	15	**3.02E-03**
**Apoptosis**	April mediated signaling	43	13	**3.72E-02**	11	9.12E-02	6	3.24E-01
	Aryl hydrocarbon receptor signaling	161	40	**1.48E-02**	34	6.92E-02	21	1.96E-01
	Myc mediated apoptosis signaling	60	20	**1.05E-02**	19	**8.71E-03**	14	**9.33E-03**
**Cellular stress and injury**	Endoplasmic reticulum stress pathway	18	7	6.03E-02	7	**3.98E-02**	4	1.73E-01
**Disease-specific pathways**	Parkinson’s signaling	16	4	4.32E-01	4	3.61E-01	-	-
**Neurotransmitters and other nervous system signaling**	Cholecystokinin/gastrin-mediated signaling	106	24	2.59E-01	20	4.56E-01	-	-

bold: with statistical significance.

## Discussion and conclusions

SUMO is a multifaceted modifier of chromatin structure. SUMO modification of chromatin proteins regulates a range of cellular processes including transcription, replication, DNA repair and chromosome segregation. SUMOylation has long been believe to be associated with gene silencing or repression. However, global mapping of chromatin binding by SUMO in yeast [7] and Drosophila [50], show that SUMOylated proteins are present at transcriptionally active and induced genes. This discovery led to the hypothesis that SUMO functions to prevent super-induction of actively transcribed genes by external factors (in this case, viral infection) to maintain a steady-state level of transcription. However, lower eukaryotes possess only one SUMO isoform, whereas there are two groups of SUMO variants in humans; SUMO-1 and SUMO-2/3. Recently, the global chromatin localization of SUMO-1 through the cell cycle of human HeLa cells has been identified. Similar to that reported in yeast, SUMO-1 tends to cluster around transcriptionally active genes [46]. Although increasing evidence from studies targeting specific cellular factors suggests that there is differential conjugation and functionality among SUMO paralogues, the global functional heterogeneity of human SUMO paralogues seems to be limited in their conjugation dynamics [11, 12] and subcellular localizations [51]. The global functional differences between SUMO paralogues in terms of epigenetic regulation remains a puzzle. In this study, we compared the chromosome-wide labeling of SUMO-1 and SUMO-2/3 proteins before and after herpesvirus reactivation using the ChIP-Seq assay. We found that firstly, on a genome-wide scale, the binding profile of the SUMO paralogues was highly similar in the control cells, but that differences were evident after KSHV reactivation with there being a significant increase in SUMO-2/3 binding while there was only limited changes in the SUMO-1 binding profile. Secondly, the distribution of both SUMO paralogues on the chromatin showed a greater tendency toward being associated with transcription regulatory regions (promoters) and that, furthermore, the binding of SUMO-2/3 onto the promoter regions was significantly increased during viral reactivation. Thirdly, there was a dramatic increase in SUMO-2/3 binding and a slight decrease in SUMO-1 binding onto TFBSs during viral reactivation. Fourthly, the potential SUMO-1 and SUMO-2/3 target TFs highly overlapped in the control cells, while the SUMO-2/3 specific TFs are significantly increased during viral reactivation. Fifthly, three IRFs, “the master regulators of immune responses” show up in the top-10 most important gene-regulating TFs targeted by SUMO-2/3 after KSHV reactivation. Sixth, both the SUMO paralogues are preferentially localized on the promoters of highly expressed genes, and that SUMO-2/3 is predominantly found associated with highly expressed genes that show no change in expression during herpesvirus reactivation. Finally, after viral reactivation, SUMO-2/3 is significantly associated with the promoters of genes in pathways related to cellular immune responses, cytokine signaling, cell growth and apoptosis. To our knowledge, our findings are the first to compare dynamically the global chromatin-binding profiles of SUMO-1 and SUMO-2/3 across the human genome and suggest that, while the binding profile of SUMO paralogues is similarly under un-induced condition, they do change differently during KSHV infection.

Herpesviruses have evolved multiple mechanisms to target SUMOylation pathways, including modulating SUMO conjugation enzymes (SUMO E1 ligase, SUMO E2 ligase and SUMO E3 ligase) and deconjugation enzymes (SUMO-specific proteases; SENP) as well as by directly targeting SUMOylated proteins [30]. Interestingly, KSHV encodes a SUMO E3 ligase in the lytic phase and this enzyme is likely to be the reason behind the increase in SUMO-2/3 paralogues present on chromatin during viral reactivation [16]. However, this hypothesis needs to be rigorously tested via a knock-in recombinant KSHV containing a SIM mutant of K-bZIP that results in a loss of its SUMO E3 ligase activity. This will be an interesting direction to investigate in the future. Moreover, we cannot exclude the possibility that the induction of K-Rta activates host SUMO E3 ligase to deposit SUMO-2/3 at the promoter regions. For example, we have previously identified a host factor, KAP1, is phosphorylated by KSHV vPK during KSHV reactivation [23] and KAP1 has recently been reported to be a SUMO E3 ligase for IRF-7 [52].

The complete sequence of the human genome was obtained more than a decade ago; nevertheless, our understanding of this genome is far from complete. The emerging concept from Encyclopedia of DNA Elements (ENCODE) is that biochemical functions of a genome can be assigned by systematically identifying the functional elements within the genome [53]. Patterns in chromatin modification or transcription factor binding onto the functional elements assists with the prediction of their role, particularly when RNA expression is examined. The global but uneven distribution of SUMO modification near TSSs prompted us to study the distribution of SUMO modification on different functional elements of the genome, such as promoters, coding sequences (transcripts), upstream gene regions, downstream gene regions, and intergenic regions. The significant enrichment of SUMO paralogues in promoter regions (Figure 2D and 2E) strongly suggests that SUMOylation may be involved in regulating gene transcription. Consistent with previous reports from lower eukaryotics and another describing SUMO-1 in HeLa cells [7, 46], the correlation between SUMO paralogues binding to promoter region and higher levels of gene transcription, which is also found in the present study (Figure 10), further supports the potential role of SUMOylation in maintaining the expression of constitutively active genes. Moreover, SUMO-1 and SUMO-2/3 may function in a similar manner maintaining the expression of transcriptional active genes in non-reactivated control cells.

SUMO binding onto chromatin must occur via either the modification of chromatin remodeling proteins or the modification of transcription factors, both of which bind to the genome. SUMO shows focal peaks or areas of high occupancy within the promoter region near TSSs. The focal and gene-selective nature of SUMO occupancy resembles the peaks associated with transcription factors, which suggests that there is SUMO modification of TFs. Motif scanning is a powerful method to facilitate the identification of DNA binding motifs (or transcription factor binding motifs) from peaks defined by ChIP-seq. This method has been widely used to distinguish the transcription regulation of one or a few TFs. SUMO modifications are able to occur in many dozens of known TFs as well as being likely to occur in many currently unknown TFs. Using the current findings, it is probably too complex and too time consuming to carrying full scale motif scanning to identify potential SUMO target TFs. Therefore, as an alternative, we used an annotation method that directly annotates SUMO peaks in the promoter region in relation to transcription factor binding sites (TFBS). The details of this method have been submitted in another article [54]. Briefly, the Transfac Matrix Database (v7.0) created by Biobase contains 258 TFBS weight matrices representing the potential DNA binding sites of 176 TFs and this database was chosen to annotate the SUMO peaks. Using this method, we were able to simultaneously identify potential SUMOylated TFs. Potential SUMO target TFs are those TFBSs that show a significant correlation with SUMO peaks and these were identified by the Hampel Identifier. Half of all SUMO-1 and half of the top-20 SUMO-2/3 potential target TFs identified before and after viral reactivation were known SUMO targets. The other half may be potential SUMO targets that have not been identified as yet or proteins containing a SIM domain that provides an additional interaction platform allowing the recruiting of other SUMOylated proteins; both of these situations may be responsible for the TFs identified here. The SUMOylation fraction in a steady state is typically very little in related to the entire pool of transcription factors. Efforts are still needed to confirm the results outlined here and to elucidate the underlying functions of SUMOylation during the regulation of these TFs. Interestingly, when we ranked the potential SUMO-2/3 target TFs by the total number of their regulating genes (Figures 4 and 5), we found three IRFs that were not SUMO-2/3 targets in the control cells that were listed as top 4th, top 5th and top 6th of the SUMO-2/3 target TFs after viral reactivation. IRFs constitute a family of TFs (IRF-1-IRF-9) that are in control of the type I interferon (IFN) system and are involved in executing the innate and adaptive immunity associated with host resistance against pathogens, including virus infection. To promote its own survival, KSHV exploits a number of different strategies to suppress the host immune system. Recent evidence has shown that the virus triggers the SUMOylation of IRFs, leading to a targeting and blocking of the type I interferon pathway [24, 40, 41]. K-bZIP of KSHV has also been found to inhibit type I IFN signaling in a signal transducers and activators of transcription (STAT) dependent manner and in an IFN-stimulated gene factor 3 (ISGF3) independent manner [39]. Moreover, KSHV K-bZIP inhibits IRF-3 by preventing IRF-3 from binding to target promoter, which precludes the formation of the enhanceosome. The potential SUMO-2/3 target IRFs identified here (Figure 5) provides an additional novel mechanism for globally inhibiting the activation of the host immune system.

The growing links between the viral and cellular SUMO systems makes SUMO a potential target for antiviral therapy [21]. Identifying the preferential usage of SUMO paralogues in viruses may help to improve the specificity of any SUMO-targeted antiviral therapies. Recently, growing evidence, including ours, suggests that some herpesviruses have a preference for SUMO-2/3 [16, 55]. Significant increase in SUMO-2/3 coating across human genome, but not in SUMO-1 coating, during viral reactivation found here suggest that a new class of combine therapy targeting SUMO-2/3 may disrupt the dynamic balance of the herpesvirus latent and lytic phases. Disrupting the balance may help the clearance of the herpesvirus from the infected cells and improve current therapy.

In summary, we found that SUMO-1 and SUMO-2/3 share a highly similar binding landscape on chromatin. They are preferentially enriched in promoter regions and are associated with highly transcribed genes. Differential chromatin-binding profiles of the SUMO paralogues are able to be observed during herpesvirus reactivation. We found that SUMO-2/3 peaks significantly increased in promoter regions during viral reactivation and this was associated with the genes that do not undergo changes in transcription level. TFs identification and GO analysis suggests that SUMO-2/3 preferentially target immune pathways during viral reactivation.

## Authors’ information

Chia-Yang Cheng is the co-first author.
